# Effects of Ocular Dominance on Contrast Sensitivity in Middle-Aged People

**DOI:** 10.1155/2014/903084

**Published:** 2014-03-09

**Authors:** Gökhan Pekel, Neşe Alagöz, Evre Pekel, Cengiz Alagöz, Ömer Faruk Yılmaz

**Affiliations:** ^1^Ophthalmology Department, Pamukkale University, Kınıklı, 20070 Denizli, Turkey; ^2^Beyoğlu Eye Research and Training Hospital, 34420 Istanbul, Turkey; ^3^Denizli State Hospital, Eye Clinic, 20125 Denizli, Turkey

## Abstract

*Purpose*. Our aim was to compare contrast sensitivity values of the dominant and nondominant eyes of healthy middle-aged subjects. *Material and Methods*. Ninety eyes of 45 healthy middle-aged subjects (30 males and 15 females) were included in this study. Patients were aged between 40 and 60 years, having uncorrected visual acuity (UCVA) of 20/25 or better (Snellen chart). Ocular dominance was determined by hole-in-the-card test. Functional acuity contrast testing (F.A.C.T.) was measured using the Optec 6500 vision testing system (Stereo Optical Co. Inc., Chicago, IL, USA) under both photopic and mesopic conditions. *Results*. At all spatial frequencies (1.5, 3, 6, 12, and 18 cpd), under mesopic conditions, the contrast sensitivity values of the dominant eyes were slightly greater than those of the nondominant eyes; but only 18 cpd spatial frequency measurements' difference was statistically significant (*P* = 0.035). Under photopic conditions, the contrast sensitivity values of the dominant eyes and non-dominant eyes were similar at all spatial frequencies (*P* > 0.05). *Conclusions*. The photopic and mesopic contrast sensitivity values of dominant and nondominant eyes of healthy middle-aged people were similar at all spatial frequencies, except at mesopic 18 cpd spatial frequency.

## 1. Introduction

Ocular dominance is the preference of one eye over the other in terms of sighting, sensory, and oculomotor tasks [1, 2]. Ocular dominance is clinically important in sports vision and vision therapy, but the most important application of the principles of ocular dominance is fitting of monovision contact lenses and applying monovision excimer laser refractive surgery for near vision [2, 3].

Contrast sensitivity is defined as the ability to differentiate between light and dark in a series of bands with no clear boundary [4]. It is revealed that impaired contrast sensitivity may be present in cases of normal visual acuity [5]. It is important to know the difference between spatial contrast sensitivity and visual acuity. Visual acuity is a measure of the spatial-resolving ability of the visual system under conditions of very high contrast (at least 85%); all targets are presented at the same contrast, but their sizes vary during the test, whereas contrast sensitivity is a measurement of the threshold contrast for seeing a target; but contrast is not kept constant during the test [6].

Contrast sensitivity is affected by various different conditions. Age and ocular dominance are some of the investigated parameters affecting contrast sensitivity. Ross et al. found that, in the age range 50–87 years when compared to 20–30 years, there was a linear decline in contrast sensitivity with age for medium and high spatial frequencies, but sensitivity for low spatial frequencies was independent of age [7]. In the aspect of ocular dominance, Suttle et al. found that interocular differences in contrast sensitivity function were not significant in most individuals [2].

Monovision therapies like contact lens for near vision, corneal refractive surgery for near vision, and multifocal intraocular lens implantation in various cases generally come into consideration in middle-aged people. Since nondominant eye is usually chosen for near vision applications, it is important to reveal whether ocular dominance affects contrast sensitivity, because monovision therapies might also have a negative influence on it. In this study, we aimed to evaluate the effects of sighting ocular dominance on contrast sensitivity in middle-aged healthy people.

## 2. Material and Methods

Ninety eyes of 45 healthy middle-aged subjects (30 males and 15 females) were included in this retrospective comparative study. The study and data collection conformed to all local laws and were compliant with the principles of the Declaration of Helsinki.

### 2.1. Study Population

Patients who were aged between 40 and 60 years and were having uncorrected visual acuity (UCVA) of 20/25 or better (Snellen chart), keratometry values between 41 and 47 Diopters, and spherical or cylindrical refractive error values between +0.50 and −0.50 Diopters were eligible for inclusion in the study. Central corneal thickness and pupil diameter measurements were also performed. Those parameters were evaluated in order to eliminate other factors that might have an influence on contrast sensitivity other than ocular dominance. Both ocular dominance and contrast sensitivity measurements were done with spectacle correction. In order to avoid observer bias, contrast sensitivity testing had been performed before ocular dominance detection.

Exclusion criteria were any ocular surgeries, ocular diseases, such as corneal opacities or irregularity, dry eye, amblyopia, anisometropia, glaucoma, retinal abnormalities, any neurological disorder, diabetes mellitus, taking medications that might affect contrast sensitivity, insufficient mental capacity to perform the tests, and any physical disability that might make it difficult to perform the test.

### 2.2. Measurement Techniques

Ocular dominance was stated by hole-in-the-card test. The participant is given a card with a small hole in the middle, instructed to hold it with both hands, and then told to view a distant object through the hole with both eyes open. The observer then alternates closing the eyes or slowly draws the opening back to the head to determine which eye is viewing the object (this is the dominant eye). This technique is used to detect sighting dominance. The test was performed three times in order to be sure.

Functional acuity contrast testing (F.A.C.T.) was measured using the Optec 6500 vision testing system (Stereo Optical Co. Inc., Chicago, IL, USA) with natural pupil under both the photopic condition (target luminance value of 85 cd/m^2^) and mesopic condition (target luminance value of 3 cd/m^2^). Sequence of testing was as follows: 1.5, 3, 6, 12, and 18 cpd (cycles per degree). Examinations under photopic conditions were done at first, and, after 10 minutes of dark adaptation, examinations under mesopic condition were performed. Contrast sensitivity values were converted to numerical values by using a conversion chart of F.A.C.T. Contrast sensitivity values of dominant and nondominant eyes were compared at all five spatial frequencies (1.5, 3, 6, 12, and 18 cpd). Since this test needs full concentration for appropriate results, all the measurements were repeated three times at all spatial frequencies. All the examinations were done by two researchers (GP and NA).

### 2.3. Statistical Analysis

For statistical analysis, SPSS 17.0 software for Windows (SPSS Inc., Chicago, IL, USA) was used. Paired samples *t*-test was used to compare contrast sensitivity values of the dominant and nondominant eyes. *P* values lower than 0.05 were considered to be statistically significant.

## 3. Results

The mean age was 51.26 (SD: 3.87) years. All the eyes had a mean UCVA of 20/25 or better. Best corrected visual acuity (BCVA) was 20/20 for all the eyes. Forty-three patients (96%) had their right eyes as dominant eye. Forty patients (91%) had their dominant eyes on the same side as the master hand.

All the eyes had keratometry values between 41 and47 Diopters. The mean central corneal thickness (CCT) of the dominant eyes was 567.04 ± 23.86 *μ*m and the mean CCT of the nondominant eyes was 563.70 ± 28.84 *μ*m (*P* = 0.21). The mean pupil diameter of the dominant eyes was 5.68 ± 0.55 mm and the mean pupil diameter of the nondominant eyes was 5.69 ± 0.54 mm (*P* = 0.75).

At all spatial frequencies (1.5, 3, 6, 12, and 18 cpd), under mesopic conditions, the contrast sensitivity values of the dominant eyes were slightly greater than those of the nondominant eyes. However, among all of the differences between the values of the two groups, only mesopic 18 cpd spatial frequency measurements were statistically significant (*P* = 0.035). Under photopic conditions, the contrast sensitivity values of the dominant eyes and nondominant eyes were similar at all spatial frequencies (*P* > 0.05).

**Figure 1:**
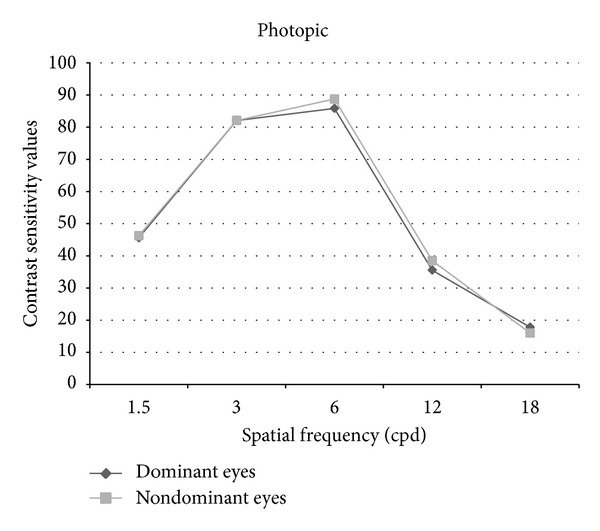
Photopic contrast sensitivity curves of the dominant and nondominant eyes.

**Figure 2:**
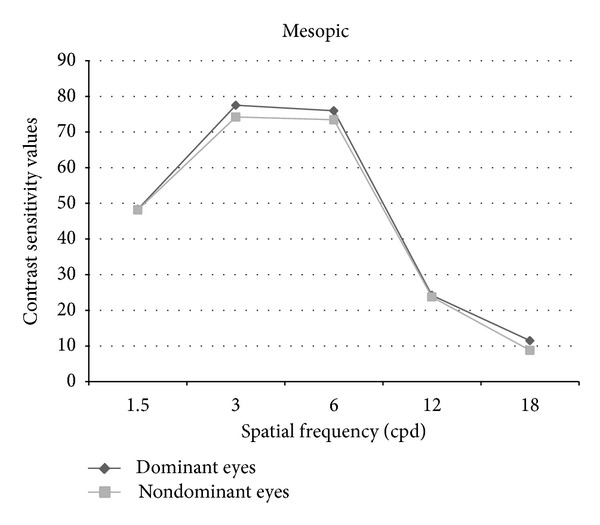
Mesopic contrast sensitivity curves of the dominant and nondominant eyes.

Figures 1 and 2 show contrast sensitivity curves of dominant and nondominant eyes. Tables 1 and 2 show numerical contrast sensitivity values and *P* values under photopic and mesopic conditions. When the data were transformed into logarithmic units, the *P* value for the comparison of dominant and nondominant eyes became 0.08 at mesopic 18 cpd. For all the other comparisons at photopic and mesopic states, *P* value was >0.05 similar to using CS scores.

## 4. Discussion

Ocular dominance is usually defined as the superiority of one eye over the other in some sensory or motor tasks. Ocular dominance is an important consideration in monovision therapies, because it helps us to decide which eye should be corrected for near or far. Ocular dominance has a strong effect on the success of monovision techniques [8]. When with both eyes open, people with normal binocular vision have no sense that one eye or the other contributes more strongly to the combined binocular vision; it is only sensed when normal binocular vision is deteriorated [9]. Generally, blurring the dominant eye causes more discomfort when compared with blurring the nondominant eye at far, so it seems convenient to choose the nondominant eye for near correction.

There are several methods to determine ocular dominance [10, 11]. In our study we applied hole-in-the-card test [12]. This is an easy, repeatable, and reliable test for sighting ocular dominance detection. The dominant eye is often, but not always, on the same side as the master hand; this statement was also acceptable for our patients.

In this study, functional acuity contrast testing (F.A.C.T.) was measured using the Optec 6500 vision testing system. In this test, the test targets were vertically oriented sine-wave gratings, so-named because the luminance of the vertical bars varied sinusoidally over space. The bar gratings presented to the test subject covered spatial frequencies of 1.5, 3, 6, 12, and 18 cycles per degree (cpd) of visual angle. In general, contrast sensitivity function had its peak sensitivity at intermediate spatial frequencies (3–6 cpd) with a steep decrease at high spatial frequencies and a more gradual decrease at lower frequencies [6].

Effects of ocular dominance on contrast sensitivity had been investigated previously to some extent. Suttle et al. found no significant interocular difference in contrast sensitivity or alignment sensitivity in their study [2]. Handa et al. investigated the effects of ocular dominance on binocular summation after monocular reading adds and found that, with strong ocular dominance, defocus results in a greater loss of contrast sensitivity than with weak ocular dominance [8]. In this study, there was not statistically significant difference at all spatial frequencies in mean photopic contrast sensitivity values between dominant and nondominant eyes. But, in mesopic condition, at 18 cpd, there was statistically significant difference that dominant eye had greater contrast sensitivity function values.

In the F.A.C.T. chart, mild refractive disorders and early cataracts generally cause contrast sensitivity losses at higher spatial frequencies, whereas severe refractive disorders and advanced cataracts cause contrast sensitivity losses at lower spatial frequencies [13, 14]. Although it was not previously reported that interocular difference occurred on higher spatial frequencies like 18 cpd, we found that dominant eye had better contrast sensitivity function values at mesopic 18 cpd spatial frequency. This result might be interpreted as deterioration of stereopsis and decrease of reading capacity (if near monovision therapy was performed) at mesopic 18 cpd.

The present study is one of the few conducted to describe contrast sensitivity function values in a middle-aged population. There are several reports in the literature about the effect of age on contrast sensitivity [15, 16]. Nomura et al. found that age-related decrease in contrast sensitivity was confirmed at all frequencies in Japanese population, even when adjusted for visual acuity, and added that contrast sensitivity tests, especially at high frequencies, assessed aspects of visual function that could not be determined in the elderly population from visual acuity tests alone [15]. Schefrin et al. revealed that statistically significant age-related declines in contrast sensitivities occurred due to age-related changes in the magnocellular pathway [16]. In this study, contrast sensitivity curves both under photopic and mesopic conditions did not show any pathological patterns in middle-aged population.

Contrast sensitivity is affected by pupil diameter [17]. Sloane et al. found that senile miosis was not responsible for older adults' loss in spatial vision; rather, older adults' miotic pupil tended to have a positive effect on their spatial vision in that it slightly improved their contrast sensitivity [17]. In this study, in order to avoid pupil diameter influences, we tried to do measurements under similar pupil diameter values.

Since monovision therapies are usually applied in middle-aged people, it is important to make visual measurements of this age group. The presbyopic patients over the age of 40 years are the best candidates for monovision [18]. Beyond the past literature, this study investigated contrast sensitivity and other ocular parameters in people whose age ranged between 40 and 60 years. Some of the factors contributing to better results in monovision therapies include good interocular blur suppression, posttreatment anisometropia of less than 2.50 Diopters, successful distance correction of the dominant eye, good stereoacuity, and motivation to adapt to this visual system [18]. Although we did not measure the degree of ocular dominance in this study, the degree of ocular dominance plays a strong role in monovision success. Handa et al. reported that patients with strong sighting preference tended to have reduced interocular blur suppression and decreased binocular depth of focus that makes monovision less tolerable [19]. Schor et al. have found that interocular blur suppression is more effective under photopic viewing conditions than high contrast mesopic and scotopic conditions [20]. Horn revealed that suppression of the out of focus image in the near eye is easier under lower contrast viewing situations [21].

Our contrast sensitivity scores provided were slightly low when compared to some other FACT data in the literature [22]. The reason might be that our patients were older. Some concerns about statistical analysis might come to one's mind that it would be better to use logarithmic units while interpreting the data; but according to us there is no definitive superiority of using logarithmic units instead of CS scores. In conclusion, in the present study, at mesopic 18 cpd, contrast sensitivity of a nondominant eye was slightly lower than that of the dominant eye. Although this parameter is not the best indicator of monovision treatment success, it is worth giving importance.

## Figures and Tables

**Table 1:** Mean photopic contrast sensitivity values of the dominant and nondominant eyes.

Spatial frequency (cpd)	Dominant eye (mean)	Nondominant eye (mean)	*P* value
1.5	45.60 ± 25.40	46.26 ± 23.86	0.76
3	82.09 ± 39.50	82.09 ± 38.63	1.00
6	85.82 ± 41.85	88.75 ± 43.65	0.56
12	35.53 ± 22.43	38.51 ± 26.25	0.20
18	17.82 ± 13.44	16.00 ± 11.55	0.15

cpd: cycles per degree.

**Table 2:** Mean mesopic contrast sensitivity values of the dominant and nondominant eyes.

Spatial frequency (cpd)	Dominant eye (mean)	Nondominant eye (mean)	*P* value
1.5	48.33 ± 24.56	48.18 ± 22.65	0.92
3	77.51 ± 37.44	74.20 ± 34.48	0.20
6	75.95 ± 39.50	73.44 ± 38.77	0.58
12	24.22 ± 18.61	23.77 ± 19.56	0.74
18	11.51 ± 10.93	8.80 ± 5.50	0.035

cpd: cycles per degree.
